# Dynamic analysis of a fuzzy Bobwhite quail population model under g-division law

**DOI:** 10.1038/s41598-024-60178-4

**Published:** 2024-04-27

**Authors:** Miao Ouyang, Qianhong Zhang, Mingji Cai, Zihao Zeng

**Affiliations:** 1 School of Mathematics and Statistics, Xiamen University of Technology, Xiamen, 361024 Fujian China; 2 School of Mathematics and Statistics, Guizhou University of Finance and Economics, Guiyang, 550025 Guizhou China; 3 School of Mathematics, Southwest Jiaotong University, Chengdu, 611756 Sichuan China

**Keywords:** Ecology, Zoology, Ecology, Mathematics and computing

## Abstract

This paper is concerned with a kind of Bobwhite quail population model

$$\begin{aligned} x_{n+1}=A+Bx_n+\frac{x_n}{x_{n-1}x_{n-2}},\ \ n=0,1,\cdots , \end{aligned}$$

where the parameters and initial values are positive parabolic fuzzy numbers. According to g-division of fuzzy sets and based on the symmetrical parabolic fuzzy numbers, the conditional stability of this model is proved. Besides the existence, boundedness and persistence of its unique positive fuzzy solution. When some fuzzy stability conditions are satisfied, the model evolution exhibits oscillations with return to a fixed fuzzy equilibrium no matter what the initial value is. This phenomena provided a vivid counterexample to Allee effect in density-dependent populations of organisms. As a supplement, two numerical examples with data-table are interspersed to illustrate the effectiveness. Our findings have been verified precise with collected northern bobwhite data in Texas, and will help to form some efficient density estimates for wildlife populations of universal applications.

## Background and motivation

Most biological phenomena use natural language and qualitative reasoning to describe ecological relationships in the description process, and artificial intelligence provides a way to process natural language knowledge, such as rule-based expert systems. In this process, knowledge is given in the form of ”IF (trigger condition) -THEN (event conclusion)”, and an ecosystem rule can be assumed as ”IF the number of species A is large and the number of species B and species C is small, THEN the number of species A increases to medium and the number of species B decreases to medium.” And the number of species C increases slowly ”, in which ”large”, ”small”, ”increase (decrease) to the medium amount” and ”slow increase” are vague and inaccurate.

To address this situation, Zadeh proposed fuzzy set theory in 1965^1^. Its core idea is to use membership function to represent fuzzy sets, membership function assigns each fuzzy object a value in the range of 0 to 1, which are classes with not sharply defined boundaries in which the transition from membership to non-membership is gradual. Fuzzy set theory provides a powerful tool for solving fuzzy expert knowledge. Fuzzy rule models composed of expert experience, fuzzy sets, fuzzy logic, etc., have been proved rational and effective for general ecosystem behavior analysis^2–4^, specially, for fishery ecological modelings^5,6^, and some epidemic prevention treatments^7,8^.

Meanwhile,a classic fuzzy set, ”$\alpha$-cut set” was proposed as a means of handling uncertainty that is due to imprecision or vagueness rather than to randomness. Algebraic structures arising out of the family of fuzzy $\alpha$-cuts and fuzzy strict ”$\alpha$-cuts” were investigated in^9^, and some significance and usefulness of fuzzy $\alpha$-cut set are discussed. Based on $\alpha$-cut sets, revealing the relationship of deterministic and uncertain models, many fuzzy models were studied worldwide. According to the $\alpha$-cut sets skills, we considered the discrete time Beverton-Holt model with fuzzy uncertainty parameters and initial conditions in^10^, and a delayed fuzzy Skellam equation in^11^, that responded to a lag between the variations of external conditions and response of the population to several variations. Meanwhile, Li and Teng^12^ studied an uncertain SIS epidemic model in 2019. More references can be sought in^13^.

Considering the biological population models, the process of habitat fragmentation has been intensified by human action of extractive, agriculture and live- stock activities. Among a habitat fragmentation, an Allee effect is an vital feature by both theoretically oriented and applied ecologists. Allee effect is a positive association between absolute average individual fitness and population size over some finite interval, as objects researched in^14^and^15,16^. However, several biology systems do not follow Allee effect in the habitats. Such as a p-fuzzy drosophila mediopunctata population system, depicted by Castanho M, which in South America Atlantic forest fragments(see^17^), exhibits oscillations with return to equilibrium. These phenomena raised doubts that the positive association of density dependence **may, but does not necessarily**, give rise to a critical population size, below which the population cannot persist. For example in 2013, Hefley, Tyre and Blankenship expected the bobwhite quail population extinct in a habitat-deteriorating and losing region with two independent data sets as in^18^. Then, our next concern is about the bobwhite quail population with two generation delays and inevitable data-errors. We will conclude that this type of population system does not affected by Allee effect, which result helps institutional to conducive ecological maintenance programs. The following is the applicable scene and development of so-called bobwhite quail population model.

In 2003, Abu-Saris et al.^19^ studied the global asymptotic stability and semicycle character of an ordinary difference equation as

$$\begin{aligned} x_{n+1}=a+\frac{x_n}{x_{n-k}}, k=2,3,4,\dots ,~~~~ n=0,1,2, \dots \end{aligned}$$

Contemporaneously, Papaschinopoulos,G et al.^20^ researched the corresponding fuzzy model,

$$\begin{aligned} x_{n+1}=A+\frac{x_n}{x_{n-m}}, m=2,3,4,\dots ,~~~~ n=0,1,2, \dots \end{aligned}$$

where $x_n$ is a sequence of fuzzy numbers, the parameter *A* is a fuzzy number. They presented the existence,boundedness and the asymptotic behavior of the positive fuzzy solutions .

Inspired by the rational difference equation system in Yang’s concern^21^, in 2005,

$$\begin{aligned} x_{n}=A+\frac{y_{n-1}}{x_{n-p} y_{n-q}}, \quad y_{n}=A+\frac{x_{n-1}}{x_{n-r} y_{n-s}}, \quad n=1,2, \ldots , \end{aligned}$$

Zhang^22^studied the following FDE in 2015. Taşdemir did so and went further in^23^, 2021.

$$\begin{aligned} x_{n+1}=A+ \frac{By_{n}}{y_{n-m}^{2}}, y_{n+1}=A+ \frac{Bx_{n}}{x_{n-m}^{2}} \end{aligned}$$

According to survey, the estimated abundance of two typical Bobwhite quails is declining by 3$\%$ per year since 1996^24^,thereby some long-term conservation efforts to the main poultry in Southern Texas are indispensable. A original bobwhite quail population model in^25^ focused only on the density of the population at spring, that is net increase, and fall, net decrease accordingly. Besides seasonal factor, living environment(brush canopy cover^26^,the effect of natural predators trap and removal^27,28^, a regular harvest^29^ ) influenced the process of bobwhite quail population evolution usually. Focusing trajectories of Bobwhite quail populations in four reasons is deemed sensible, our observations can be expressed in a generalization model of a form originally introduced by^30–32^ as

$$\begin{aligned} f\left( u_{n}\right) =a+b u_{n}+\frac{c u_{n}}{1+u_{n}^{d}}, \quad a, b\ge 0, c, d > 0 \end{aligned}$$

According to Zhang and Taşdemir’s work, the following fuzzy difference equation, typically and not unexpectedly, described a general fuzzy bobwhite quail population model (GFBQP model).

1 $$\begin{aligned} x_{n+1}=A+Bx_{n}+\frac{C x_{n}}{D+\prod _{k=1}^{m} x_{n-k}^{p_{k}}}, \quad p_k,C~>~0,~~ A, B, D \ge 0, ~~~ m={1,2,\dots } \end{aligned}$$

This paper simplified above model with $C=1, ~D=0, ~m=2, ~p_1=p_2=1$, saying a fuzzy bobwhite quail population (FBQP) model

2 $$\begin{aligned} x_{n+1}=A+B x_{n}+\frac{x_{n}}{x_{n-1} x_{n-2}}, \quad n=0,1, \cdots , \end{aligned}$$

where the initial population size values $x_{i}, i=-2,-1,0$, and parameters *A* , that indicates some natural logarithm item of process error term during population-size change, see^33^ , and *B*, that indexes population threshold density^25^, are positive fuzzy numbers.

We proposed and studied the behavior of positive fuzzy solutions of Eq. (2), applying $\alpha$- cut sets and g-division(more natural to understand than Zadeh Extension principle, as in^34^.

This article is mainly to investigate the dynamical behaviors of a third-order fuzzy Bobwhite quail populations Model. The content of this paper is organized as follows. Section 2 introduced the related terms and definitions. Section 3 proposes the main theorems and proofs including existence, boundedness, persistence and asymptomatic stability of positive fuzzy solutions under some sufficient conditions. A unique positive fuzzy equilibrium *x* and every positive fuzzy solution $x_n$ of Eq. (2) also was drawn to converges to *x* as $n\rightarrow \infty$. Section 4 presents the numerical results for two test problems in parabolic fuzzy number^35^, which is an upgraded vision of triangle fuzzy number, and is well-adapted for application more information and application can refer^36,37^ and^38^. The conclusion of the article is presented in Section 5.

## Some definitions

Firstly we give some definitions will be used in the following.

### Definition 2.1

^39^ A function $H: R\rightarrow [0,1]$ is called a fuzzy number if the following conditions (i)-(iv) hold true:

(i) *H* is normal, namely, there is at least an $x\in R$ satisfying $H(x)=1$;
(ii) *H* is fuzzy convex, namely, for each $\lambda \in [0,1]$ and $x_1, x_2\in R$, it has
  $$\begin{aligned} H(\lambda x_1+(1-\lambda )x_2)\ge \min \{H(x_1),H(x_2)\}; \end{aligned}$$
(iii) *H* is upper semi-continuous;
(iv) The support of *H*, $\text{ supp }H=\overline{\bigcup _{\alpha \in (0,1]}[H]_{\alpha }}=\overline{\{x: H(x)>0\}}$ is compact.

The $\alpha$-level set of fuzzy number *H* is written $[H]_\alpha =\{x\in R: H(x)\ge \alpha \}$ for $\alpha \in (0,1]$. It is clear that $[H]_\alpha$ is a closed interval. *H* is positive (or negative) if $\text{ supp } H\subset (0,+\infty ) (\text{ supp } H\subset (-\infty ,0) ).$ If *H* is a positive real number (trivial fuzzy number), then $[H]_\alpha =[H, H],$ for $\alpha \in (0,1].$

Let *H*, *P* be fuzzy numbers with $\alpha$ level set $[H]_\alpha =[H_{l,\alpha }, H_{r,\alpha }], [P]_\alpha =[P_{l,\alpha }, P_{r,\alpha }],\alpha \in [0,1]$, the addition and multiplication of fuzzy numbers are defined as follows:

3 $$\begin{aligned}{}[H+P]_\alpha =[H_{l,\alpha }+P_{l,\alpha },H_{r,\alpha }+P_{r,\alpha }], \end{aligned}$$

4 $$\begin{aligned}{}[kH]_\alpha =[kH_{l,\alpha },kH_{r,\alpha }], k>0. \end{aligned}$$

The collection of all fuzzy numbers satisfying Eqs.(2.1)-(2.2) is denoted by $R_F$.

### Definition 2.2

^39^ The metric *D* between arbitrary two fuzzy numbers *H* and *P* is denoted by

$$\begin{aligned} D(H,P)=\sup _{\alpha \in [0,1]}\max \{|H_{l,\alpha }-P_{l,\alpha }|,|H_{r,\alpha }-P_{r,\alpha }|\}. \end{aligned}$$

It is obvious that $(R_F, D)$ forms a complete metric space.

### Definition 2.3

^40^ Let $H,P\in R_F$, $[H]_\alpha =[H_{l,\alpha }, H_{r,\alpha }], [P]_\alpha =[P_{l,\alpha }, P_{r,\alpha }]$, with $0\notin [P]_\alpha , \forall \alpha \in [0,1]$. The g-division ($\div _g$) is denoted by $W=H\div _g P$ having level sets $[W]_\alpha =[W_{l,\alpha },W_{r,\alpha }]$(here $[H]_\alpha ^{-1}=[1/H_{r,\alpha },1/H_{l,\alpha }]$)

$$\begin{aligned}{}[W]_\alpha =[H]_\alpha \div _g[P]_\alpha \Longleftrightarrow \left\{ \begin{array}{ll} (i) &{} [H]_\alpha =[P]_\alpha [W]_\alpha ,\\ \text{ or }&{}\\ (ii)&{} [P]_\alpha =[H]_\alpha [W]_\alpha ^{-1}. \end{array} \right. \end{aligned}$$

If *W* is a proper fuzzy number, i.e., $W_{l,\alpha }$ and $W_{r,\alpha }$ are nondecreasing and nonincreasing respectively, and $W_{l,1}\le W_{r,1}$.

Compared with utilizing Zadeh extension principle, g-division introduced in^40^ has an obvious advantage that it decreases the singularity of fuzzy solution due to reduction of the length of the support interval. The g-division reduced some negligible ambiguity degree, is superior to the Zadeh Extension principle in fuzzy number operations. The g-division is the logic basis of several analysis methods, for example, Fanny method was considered to be one of the best choices in^41^, because it produced the largest reductions in the variance of three fields cultivated with soya bean and maize in Brazil. It is utilized by us in^42^ to present large time behaviors of positive fuzzy solution of a kind of second-order fractal difference equation with positive fuzzy parameters, including persistence, boundedness, global convergence.

### Remark 2.1

In this paper, according to^40^, if the positive fuzzy number $H\div _g P=W\in R_F$ exists, then one and only one of following two cases will be held.

Case I   if $H_{l,\alpha }P_{r,\alpha }\le H_{r,\alpha }P_{l,\alpha },\forall \alpha \in [0,1],$ then $W_{l,\alpha }=\frac{H_{l,\alpha }}{P_{l,\alpha }}, W_{r,\alpha }=\frac{H_{r,\alpha }}{P_{r,\alpha }},$

Case II  if $H_{l,\alpha }P_{r,\alpha }> H_{r,\alpha }P_{l,\alpha },\forall \alpha \in [0,1],$ then $W_{l,\alpha }=\frac{H_{r,\alpha }}{P_{r,\alpha }}, W_{r,\alpha }=\frac{H_{l,\alpha }}{P_{l,\alpha }}.$

### Definition 2.4

Let $\{x_n\}$ be a sequence of positive fuzzy number, if there exists a $M>0$, resp. $N>0$, satisfying

$$\begin{aligned} \text{ supp }\ x_n\subset [M,\infty ), n=1,2,\cdots ,\ \ \text{ resp }.\ \text{ supp }\ x_n\subset (0,N],n=1,2,\cdots \end{aligned}$$

then $\{x_n\}$ is persistent, resp. bounded.

If there exist $M, N>0$ such that

$$\begin{aligned} \text{ supp }\ x_n\subset [M,N], n=1, 2, \cdots . \end{aligned}$$

then the sequence $\{x_n\}$ is bounded and persistent.

If the norm $\Vert x_n\Vert ,n=1,2,\cdots ,$ is an unbounded sequence, then the sequence $\{x_n\}$ is unbounded.

### Definition 2.5

$x_n$ is said to be a positive solution of Eq. (2) if a sequence $\{x_n\}$ satisfies Eq. (2). *x* is a positive equilibrium of Eq. (2) if

$$\begin{aligned} x=A+Bx+\frac{x}{x^2}. \end{aligned}$$

If $\lim _{n\rightarrow \infty }D(x_n,x)=0$, then $\{x_n\}$ converges to *x* as $n\rightarrow \infty$.

## Main results and its proof

### Existence of a unique solution of equation (2)

Firstly, we propose the lemma of multi-variable fuzzy function with $\alpha$-cut set.

#### Lemma 3.1

^39^ Let $g: R^+\times R^+\times R^+\times R^+\rightarrow R^+$ be continuous, $A_i\in R_F^+, i=1,2,3,4$, then

$$\begin{aligned}{}[g(A_1,A_2,A_3,A_4)]_\alpha =g([A_1]_\alpha ,[A_2]_\alpha ,[A_3]_\alpha ,[A_4]_\alpha ),\ \ \alpha \in (0,1]. \end{aligned}$$

#### Theorem 3.1

Consider Eq. (2), where coefficients $A, B\in R_F^+$ and $x_i\in R_F^+,i=-2,-1, 0$. Then there is a unique positive fuzzy solution $x_n$ of Eq. (2).

#### Proof

Assume that a sequence of fuzzy numbers $\{x_n\}$ is satisfied with Eq. (2) for initial conditions $x_i\in R_F^+,i=-2,-1,0.$ Consider the $\alpha$-level set, $\alpha \in (0,1],$

5 $$\begin{aligned}{}[x_n]_\alpha =[x_{n,l,\alpha },x_{n,r,\alpha }], \ \ [A]_\alpha =[A_{l,\alpha }, A_{r,\alpha }], \ \ [B]_\alpha =[B_{l,\alpha }, B_{r,\alpha }], \ \ n=0,1,2,\cdots . \end{aligned}$$

By virtue of (3.1) and Lemma 3.1, taking $\alpha -$level set, it follows from Eq. (2) that

$$\begin{aligned}{}[x_{n+1}]_\alpha= & {} [x_{n+1,l,\alpha },x_{n+1,r,\alpha }]\\= & {} \left[ A+Bx_n+\frac{x_n}{x_{n-1}x_{n-2}}\right] _\alpha =[A]_\alpha +[B]_\alpha \times [x_n]_\alpha +\frac{[x_n]_\alpha }{[x_{n-1}]_\alpha \times [x_{n-2}]_\alpha }\\= & {} [A_{l,\alpha },A_{r\alpha }]+[B_{l,\alpha }x_{n,l,\alpha },B_{r,\alpha }R_{n,\alpha }] +\frac{[x_{n,l,\alpha },x_{n,r,\alpha }]}{[x_{n-1,r,\alpha }x_{n-2,r,\alpha }, x_{n-1,r,\alpha }x_{n-2,r,\alpha }]}\\= & {} [A_{l,\alpha }+B_{l,\alpha }x_{n,l,\alpha },A_{r,\alpha }+B_{r,\alpha }x_{n,r,\alpha }]+ {\frac{[x_{n,l,\alpha },x_{n,r,\alpha }]}{[x_{n-1,l,\alpha }x_{n-2,r,\alpha }, x_{n-1,r,\alpha }x_{n-2,r,\alpha }]}, } \end{aligned}$$

According to g-division, we have two cases. $\square$

Case I

6 $$\begin{aligned}{}[x_{n+1}]_\alpha= & {} [x_{n+1,l,\alpha },x_{n+1,r,\alpha }]\nonumber \\= & {} \left[ A_{l,\alpha }+B_{l,\alpha }x_{n,l,\alpha }+\frac{x_{n,l,\alpha }}{x_{n-1,l,\alpha }x_{n-2,l,\alpha }}, A_{r,\alpha }+B_{r,\alpha }x_{n,r,\alpha }+\frac{x_{n,r,\alpha }}{x_{n-1,r,\alpha }x_{n-2,r,\alpha }}\right] , \end{aligned}$$

Case II

7 $$\begin{aligned}{}[x_{n+1}]_\alpha= & {} [x_{n+1,l,\alpha },x_{n+1,r,\alpha }]\nonumber \\= & {} \left[ A_{l,\alpha }+B_{l,\alpha }x_{n,l,\alpha }+\frac{x_{n,r,\alpha }}{x_{n-1,r,\alpha }x_{n-2,r,\alpha }}, A_{r,\alpha }+B_{r,\alpha }x_{n,r,\alpha }+\frac{x_{n,l,\alpha }}{x_{n-1,l,\alpha }x_{n-2,l,\alpha }}\right] . \end{aligned}$$

If Case I occurs, for $n\in \{0,1,2,\cdots \}, \alpha \in (0,1]$, it follows from (3.2) that

8 $$\begin{aligned}{} & {} x_{n+1,l,\alpha }=A_{l,\alpha }+B_{l,\alpha }x_{n,l,\alpha }+\frac{x_{n,l,\alpha }}{x_{n-1,l,\alpha }x_{n-2,l,\alpha }},\nonumber \\{} & {} R_{n+1,\alpha }=A_{r,\alpha }+B_{r,\alpha }x_{n,r,\alpha }+\frac{x_{n,r,\alpha }}{x_{n-1,r,\alpha }x_{n-2,r,\alpha }}. \end{aligned}$$

Then, for each initial values $(x_{j,l,\alpha },x_{j,r,\alpha }), j=-2, -1,0,\alpha \in (0,1]$, there is a unique solution $x_{n,\alpha }$.

Now we show that $x_{n,\alpha },\alpha \in (0,1]$, ascertains the fuzzy solution of Eq. (2) with initial values $x_i,i=-2,-1,0$ satisfying

9 $$\begin{aligned}{}[x_n]_\alpha =[x_{n,l,\alpha },x_{n,r,\alpha }],\ \ n\in \{0,1,2,\cdots \}, \ \alpha \in (0,1]. \end{aligned}$$

Since $x_j\in R_F^+, j=-2, -1,0$, It follows from reference^19^ that, for any $\alpha _i\in (0,1] (i=1,2),\alpha _1\le \alpha _2$,

10 $$\begin{aligned} 0<x_{j,l,\alpha _1}\le x_{j,l,\alpha _2}\le x_{j,r,\alpha _2}\le x_{j,r,\alpha _1},\ \ j=0, -1, -2. \end{aligned}$$

Firstly, we prove that, for $n=0,1,2,\cdots ,$

11 $$\begin{aligned} x_{n,l,\alpha _1}\le x_{n,l,\alpha _2}\le x_{n,r,\alpha _2}\le x_{n,r,\alpha _1}. \end{aligned}$$

Since (3.6) hold true, (3.7) is true by mathematical induction for $n=0$. When $n= k, k\in \{1,2,\cdots \}$, Let (3.7) be true. Then, for $n=k+1$, it follows from (3.5)-(3.7) that

$$\begin{aligned} x_{k+1,l,\alpha _1}= & {} A_{l,\alpha _1}+B_{l,\alpha _1}x_{k,l,\alpha _1}+\frac{x_{k,l,\alpha _1}}{x_{k-1,l,\alpha _1}x_{k-2,l,\alpha _1}} \le A_{l,\alpha _2}+B_{l,\alpha _2}x_{k,l,\alpha _2}+\frac{x_{k,l,\alpha _2}}{x_{k-1,l,\alpha _2}x_{k-2,l,\alpha _2}}=x_{k+1,l,\alpha _2}\\= & {} A_{l,\alpha _2}+B_{l,\alpha _2}x_{k,l,\alpha _2}+\frac{x_{k,l\alpha _2}}{x_{k-1,l,\alpha _2}x_{k-2,l,\alpha _2}}\le A_{r,\alpha _2}+B_{r,\alpha _2}x_{n,r,\alpha _2} +\frac{x_{k,r,\alpha _2}}{x_{k-1,r,\alpha _2}x_{k-2,r,\alpha _2}}=x_{k+1,r,\alpha _2}\\= & {} A_{r,\alpha _2}+B_{r,\alpha _2}x_{k,r,\alpha _2}+\frac{x_{k,r,\alpha _2}}{x_{k-1,r,\alpha _2}x_{k-2,r,\alpha _2}}\le A_{r,\alpha _1}+B_{r,\alpha _1}x_{k,r,\alpha _1}+\frac{x_{k,r,\alpha _1}}{x_{k-1,r,\alpha _1}x_{k-2,r,\alpha _1}}=x_{k+1,r,\alpha _1} \end{aligned}$$

Therefore, (3.7) is true.

From (3.5), we know

12 $$\begin{aligned}{} & {} x_{1,l,\alpha }=A_{l,\alpha }+B_{l,\alpha }x_{0,l,\alpha }+\frac{x_{0,l,\alpha }}{x_{-2,l,\alpha }x_{-1,l,\alpha }},\nonumber \\{} & {} x_{1,r,\alpha }=A_{r,\alpha }+B_{r,\alpha }x_{0,r,\alpha }+\frac{x_{0,r,\alpha }}{x_{-2,r,\alpha }x_{-1,r,\alpha }},\ \ \alpha \in (0,1]. \end{aligned}$$

Since $x_j\in R_F^+, j=-2, -1, 0,$ and $A, B\in R_F^+$, it follows that $x_{j,l,\alpha },x_{j,r,\alpha }, j=0,-1,-2,$ are left continuous.

Therefore, it follows from (3.8) that $x_{1,l,\alpha }$ and $x_{1,r,\alpha }$ are left continuous. So, it’s natural by induction that $x_{n,l,\alpha }$ and $x_{n,r,\alpha }$are left continuous

Secondly, it is sufficient that $\text{ supp } x_n=\overline{\bigcup _{\alpha \in (0,1]}[x_{n,l,\alpha },x_{n,r,\alpha }]}$ is compact, namely, $\bigcup _{\alpha \in (0,1]}[x_{n,l,\alpha },x_{n,r,\alpha }]$ is bounded.

Let $n=1$, since $A, B\in R_F^+$ and $x_j\in R_F^+,j=-2, -1, 0$, for each $\alpha \in (0,1]$, there are positive real numbers $A_{l,0},A_{r,0},B_{l,0},B_{r,0}, x_{j,l,0}, x_{j,r,0},j=-2,-1,0,$ such that

13 $$\begin{aligned}{}[A_{l,\alpha },A_{r,\alpha }]\subset [A_{l,0},A_{r,0}],\ \ [B_{l,\alpha },B_{r,\alpha }]\subset [B_{l,0},B_{r,0}],\ \ \ [x_{j,l,\alpha },x_{j,r,\alpha }]\subset [x_{j,l,0}, x_{j,r,0}]. \end{aligned}$$

Hence from (3.8) and (3.9), it has

$$\begin{aligned}{}[x_{1,l,\alpha },x_{1,r,\alpha }]\subset \left[ A_{l,0}+B_{l,0}x_{j,l,0} +\frac{x_{0,l,0}}{x_{-1,l,0}x_{-2,l,0}},A_{r,0}+B_{r,0}x_{j,r,0}+\frac{x_{0,r,0}}{x_{-1,r,0}x_{-2,r,0}}\right] ,\alpha \in (0,1]. \end{aligned}$$

Then

14 $$\begin{aligned} \bigcup _{\alpha \in (0,1]}[x_{1,l,\alpha },x_{1,r,\alpha }]\subset \left[ A_{l,0}+B_{l,0}x_{0,l,0}+\frac{x_{0,l,0}}{x_{-1,l,0}x_{-2,l,0}}, A_{r,0}+B_{l,0}x_{0,r,0}+\frac{x_{0,r,0}}{x_{-1,r,0}x_{-2,r,0}}\right] . \end{aligned}$$

Therefore, $\overline{\bigcup _{\alpha \in (0,1]}[x_{1,l,\alpha }, x_{1,r,\alpha }]}\subset (0,\infty )$ is compact.

Deducing inductively, it is easy to get that $\overline{\bigcup _{\alpha \in (0,1]}[x_{n,l,\alpha }, x_{n, r,\alpha }]}$ is compact, and

15 $$\begin{aligned} \overline{\bigcup _{\alpha \in (0,1]}[x_{n,l,\alpha }, x_{n,r,\alpha }]}\subset (0,\infty ), \ \ n=1,2,\cdots . \end{aligned}$$

Noting (3.7) and (3.11), $x_{n,l,\alpha },$ and $x_{n, r,\alpha }$ are left continuous, $[x_{n,l,\alpha },x_{n,r,\alpha }]$ ascertains a sequence of positive fuzzy numbers $x_n$ satisfying (3.5).

Now we show that $x_n$ is the positive fuzzy solution of Eq. (2) with the initial conditions $x_i, i=0,-1,-2$. For $\alpha \in (0,1]$,

$$\begin{aligned}{}[x_{n+1}]_\alpha= & {} [x_{n+1,l,\alpha },x_{n+1,r,\alpha }]\\= & {} \left[ A_{l,\alpha }+B_{l,\alpha }x_{n,l,\alpha }+\frac{x_{n,l,\alpha }}{x_{n-1,l,\alpha }x_{n-2,l,\alpha }}, A_{r,\alpha }+B_{r,\alpha }x_{n,r,\alpha }+\frac{x_{n,r,\alpha }}{x_{n-1,r,\alpha }x_{n-2,r,\alpha }}\right] \\= & {} \left[ A+Bx_n+\frac{x_{n}}{x_{n-1}x_{n-2}}\right] _\alpha , \end{aligned}$$

We deduce $x_n$ is a positive fuzzy solution of Eq. (2) with initial values $x_i, i=-2,-1,0$.

If there is another positive fuzzy solution ${\overline{x}}_n$ of Eq. (2) with initial values $x_i, i=-2, -1, 0$, it is easy to show that

16 $$\begin{aligned}{}[{\overline{x}}_n]_\alpha =[x_{n,l,\alpha }, x_{n,r,\alpha }],\ \ \ \alpha \in (0,1],\ \ \ n=0,1,2,\cdots . \end{aligned}$$

From (3.5) and (3.12), then $[x_n]_\alpha =[{\overline{x}}_n]_\alpha , \alpha \in (0,1],n=0,1,2,\cdots ,$ so $x_n={\overline{x}}_n, n=0,1,\cdots .$

Suppose Case II occurs, for $n\in \{0,1,2,\cdots \}, \alpha \in (0,1]$, it follows from (3.3) that

17 $$\begin{aligned}{} & {} L_{n+1,\alpha }=A_{l,\alpha }+B_{l,\alpha }x_{n,l,\alpha }+\frac{x_{n,l,\alpha }}{x_{n-1,l,\alpha }x_{n-2,l,\alpha }}, \nonumber \\{} & {} R_{n+1,\alpha }=A_{r,\alpha }+B_{r,\alpha }x_{n,r,\alpha }+\frac{x_{n,r,\alpha }}{x_{n-1,r,\alpha }x_{n-2,r,\alpha }}. \end{aligned}$$

The proof is similar to those of Case I. Thus we finish the proof of Theorem 3.1.

### Dynamics of equation (2)

In this section, by virtue of g-division of fuzzy numbers, we investigate the dynamical behavior of the positive fuzzy solutions of Eq. (2) by cases I and cases II.

**Firstly, if case I occurs, **we draw a conclusion of corresponding crisp system in the following lemma.

#### Lemma 3.2

Consider the following difference equation

18 $$\begin{aligned} y_{n+1}=p+ay_n+\frac{y_n}{y_{n-1}y_{n-2}},\ \ n=0,1,\cdots , \end{aligned}$$

where $$y_i>0, \ \ i=-2,-1,0$$, if

19 $$\begin{aligned} \left\{ \begin{array}{l} 0~<~a~<~1,\\ \\ (1-a)p^{2}~>~1, \end{array} \right. \end{aligned}$$

then for $$n\ge 0$$

20 $$\begin{aligned} p< y_n< \frac{p^3}{(1-a)p^2-1}+y_3. \end{aligned}$$

#### Proof

From (3.14) it is clear that $y_n>p$ for $n\ge 1$ . For $n\ge 4$, one can get that

21 $$\begin{aligned} y_n=p+ay_{n-1}+\frac{y_{n-1}}{y_{n-2}y_{n-3}}\le p+\frac{1+ap^2}{p^2}y_{n-1}. \end{aligned}$$

Deducing inductively, for $n-k\ge 3$, it follows that

$$\begin{aligned} y_n\le & {} p+p\frac{1+ap^2}{p^2}+\left( \frac{1+ap^2}{p^2}\right) ^2y_{n-2}\le p+p\frac{1+ap^2}{p^2}+p\left( \frac{1+ap^2}{p^2}\right) ^2+\left( \frac{1+ap^2}{p^2}\right) ^3y_{n-3}\nonumber \\\le & {} p+p\frac{1+ap^2}{p^2}+p\left( \frac{1+ap^2}{p^2}\right) ^2+p\left( \frac{1+ap^2}{p^2}\right) ^3 +\left( \frac{1+ap^2}{p^2}\right) ^4y_{n-4}\nonumber \\\le & {} \cdots \le \sum _{i=1}^{k}p\left( \frac{1+ap^2}{p^2}\right) ^{i-1}+\left( \frac{1+ap^2}{p^2}\right) ^ky_{n-k}\nonumber \\= & {} \frac{p}{1-a-1/p^2}\left[ 1-\left( \frac{1+ap^2}{p^2}\right) ^{k}\right] +\left( \frac{1+ap^2}{p^2}\right) ^ky_{n-k}\nonumber \\\le & {} \frac{p^3}{(1-a)p^2-1}+y_{n-k}, \end{aligned}$$

$$\square $$

Noting that $k\le n-3$ is equivalent to $n-k\ge 3$. So (3.16) is true.

By Lemma 3.2, the following theorem interprets the sufficient conditions for the positive fuzzy solution $x_n$ of Eq. (2) will be bounded and persistent.

#### Theorem 3.2

Consider Eq. (2), where the parameters $A, B\in R_F^+$ and the initial conditions $x_i\in R_F^+, i=-2, -1, 0$, if

22 $$\begin{aligned} \left\{ \begin{array}{l} A_{r,\alpha }\le A_{r,0}<1,\\ \\ (1-B_{l,\alpha })A_{l,\alpha }^2>1, \\ \\ (1-B_{r,\alpha })A_{r,\alpha }^2>1,\\ \\ x_{n,l,\alpha }x_{n-1,r,\alpha }x_{n-2,r,\alpha } \le x_{n,r,\alpha }x_{n-1,l,\alpha }x_{n-2,l,\alpha }, \end{array} \right. \end{aligned}$$

then every positive fuzzy solution $x_n$ of Eq. (2) is bounded and persistent.

#### Proof

(i) Let $x_n$ be a positive solution of Eq. (2) satisfying (3.5). It follows from (3.4) that

23 $$\begin{aligned} x_{n,l,0}\le x_{n,l,\alpha },\ \ x_{n,l,0}\le x_{n,r,\alpha }, \ \ n=1,2,\cdots ,\ \ \alpha \in (0,1]. \end{aligned}$$

Then from (3.9), (3.18) and Lemma 3.2, we get from(3.1) and (3.4)

24 $$\begin{aligned}{}[x_{n,l,\alpha },x_{n,r,\alpha }]\subset [A_{l,0},\frac{A_{l,0}^3}{(1-B_{l,0})A_{l,0}^2-1}+x_{3,l,0}] \times [ A_{r,0},\frac{A_{r,0}^3}{(1-B_{r,0})A_{r,0}^2-1}+x_{3,r,0}],\ \ n\ge 5, \end{aligned}$$

$$\square $$

Theorem 3.2 reveals the relation between the population development error and the population threshold density to guarantee a FBQP model steady, when initial size meets Case I.

#### Lemma 3.3

Consider difference equation (3.14), if

25 $$\begin{aligned} \left\{ \begin{array}{l} 0< a< 1,\\ \\ 3p^2 >4(1-a), \end{array} \right. \end{aligned}$$

then Eq. (3.14) is asymptotically stable, and its equilibrium point is ${\bar{y}}=\frac{p+\sqrt{p^2+4(1-a)}}{2(1-a)}$.

#### Proof

It is easy to obtain the equilibrium point ${\bar{y}}$ of (3.14). Considering the linearized equation of (3.14) on ${\overline{y}}$, by the methodologies in^43–45^ associated with (3.14), is

26 $$\begin{aligned} y_{n+1}-\left[ a+G\right] y_n+Gy_{n-1}+Gy_{n-2}=0,\ \ n=0,1,2,\cdots , \end{aligned}$$

where $$G=\frac{2(1-a)^2}{p^2+2(1-a)+p\sqrt{p^2+4(1-a)}}.$$

Since $3p^2 > 4(1-a)$, it leads

$$\begin{aligned} \frac{6(1-a)}{p^2+2(1-a)+p\sqrt{p^2+4(1-a)}}<1. \end{aligned}$$

Using Theorem 1.3.7 in^43^, the equilibrium ${\overline{y}}$ of (3.14) is asymptotically stable. $\square$

#### Lemma 3.4

Consider the system of ordinary difference equations in Case I

27 $$\begin{aligned} y_{n+1}=p+ay_n+\frac{y_n}{y_{n-1}y_{n-2}},\ \ z_{n+1}=q+bz_n+\frac{z_n}{z_{n-1}z_{n-2}},\ \ n=0,1,\cdots , \end{aligned}$$

if

28 $$\begin{aligned} 0< a<1,~~ 0< b< 1,~~ 3p^2>4(1-a), ~~ 3q^2>4(1-b), \end{aligned}$$

Then every positive solution $(y_n,z_n)$ of (3.24) tends to equilibrium

29 $$\begin{aligned} ({\overline{y}},{\overline{z}})=\left( \frac{p+\sqrt{p^2+4(1-a)}}{2(1-a)}, \frac{q+\sqrt{q^2+4(1-b)}}{2(1-b)}\right) . \end{aligned}$$

#### Proof

Let $(y_n,z_n)$ be positive solution of (3.24). Set

$$\begin{aligned} \Lambda _1=\lim _{n\rightarrow \infty }\sup y_n,\ \ \lambda _1=\lim _{n\rightarrow \infty }\inf y_n, \Lambda _2=\lim _{n\rightarrow \infty }\sup z_n,\ \ \lambda _2=\lim _{n\rightarrow \infty }\inf z_n. \end{aligned}$$

From Lemma 3.2, we have $0<p<\lambda _1\le {\overline{y}}\le \Lambda _1<\infty , 0<q<\lambda _2\le {\overline{z}}\le \Lambda _2<\infty .$ Then

30 $$\begin{aligned} \Lambda _1\le p+a\Lambda _1+\frac{\Lambda _1}{\lambda _1{\overline{y}}}, \ \ \Lambda _2\le q+b\Lambda _2+\frac{\Lambda _2}{\lambda _2{\overline{z}}}, \ \ \lambda _1\ge p+a\lambda _1+\frac{\lambda _1}{\Lambda _1{\overline{y}}}, \ \ \lambda _2\ge q+b\lambda _2+\frac{\lambda _2}{\Lambda _2{\overline{z}}}. \end{aligned}$$

Relations (3.27) implies that

$$\begin{aligned} {\bar{y}}p\Lambda _1+\lambda _1\le {\overline{y}}p\lambda _1+\Lambda _1,\ \ {\overline{z}}q\Lambda _2+\lambda _2\le {\overline{z}}q\lambda _2+\Lambda _2. \end{aligned}$$

That is

31 $$\begin{aligned} ({\overline{y}}p-1)(\Lambda _1-\lambda _1) \le 0, \ ({\overline{z}}q-1)(\Lambda _2-\lambda _2) \le 0. \end{aligned}$$

Since condition (3.25) hold, we can get

$$\begin{aligned} {\overline{y}}p> 1,\ \ \ {\overline{z}}q > 1. \end{aligned}$$

Since $\lambda _1\le \Lambda _1,\lambda _2\le \Lambda _2$, then from (3.28), it is obvious that

$$\begin{aligned} \lambda _1 = \Lambda _1,\ \ \lambda _2 = \Lambda _2. \end{aligned}$$

Thus $\lim _{n\rightarrow \infty }y_n$ and $\lim _{n\rightarrow \infty }z_n$ exist, referring^46^. From the uniqueness of the positive equilibrium of (3.14), we have that $\lim _{n\rightarrow \infty }y_n={\overline{y}}, \ \lim _{n\rightarrow \infty }z_n={\overline{z}}.$ $\square$

#### Theorem 3.3

For $$\alpha \in (0,1],$$ $$A \in R_F^+, B \in R^+$$, if

32 $$\begin{aligned} \left\{ \begin{array}{l} A_{r,\alpha }\le A_{r,0}<1,~~\\ \\ A_{r,\alpha }B_{l,\alpha }-A_{l,\alpha }B_{r,\alpha }<A_{r,\alpha }-A_{l,\alpha }\\ \\ 3A_{l,\alpha }^2>4(1-B_{l,\alpha }), ~~ 3A_{r,\alpha }^2>4(1-B_{r,\alpha }),\\ \\ x_{n,l,\alpha }x_{n-1,r,\alpha }x_{n-2,r,\alpha } \le x_{n,r,\alpha }x_{n-1,l,\alpha }x_{n-2,l,\alpha }, \end{array} \right. \end{aligned}$$

then every positive solution $x_n$ of Eq. (2) converges to the positive equilibrium *x*, where $[x]_\alpha =[x_{l,\alpha }, x_{r,\alpha }],$

33 $$\begin{aligned} x_{l,\alpha }=\frac{A_{l,\alpha }+\sqrt{A_{l,\alpha }^{2}+4(1-B_{l,\alpha })}}{2(1-B_{l,\alpha })},~~~ x_{r,_\alpha }=\frac{A_{r,\alpha }+\sqrt{A_{r,\alpha }^{2}+4(1-B_{r,\alpha })}}{2(1-B_{r,\alpha })}, \end{aligned}$$

and $$\lim _{n\rightarrow \infty }D(x_n,x)=0$$.

#### Proof

Suppose that there is a positive fuzzy number *x* satisfying

$$\begin{aligned} x=A+Bx+\frac{x}{x^2},\ \ [x]_\alpha =[x_{l,\alpha },x_{r,\alpha }],\ \ \alpha \in (0,1]. \end{aligned}$$

where $$x_{l,\alpha }, x_{r,\alpha }\ge 0$$. Then

$$\begin{aligned}{} & {} x_{l,\alpha }=A_{l,\alpha }+Bx_{l,\alpha }+\frac{x_{l,\alpha }}{x_{l,\alpha }^2},\nonumber \\{} & {} x_{r,\alpha }=A_{r,\alpha }+Bx_{r,\alpha }+\frac{x_{r,\alpha }}{x_{r,\alpha }^2}. \end{aligned}$$

it gets (3.30)

Let $x_n$ be a positive solution of Eq. (2). Since(3.29), it follows from system (3.4), by Lemma 3.3 and Lemma 3.4, that

$$\begin{aligned} \lim _{n\rightarrow \infty }x_{n,l,\alpha }=x_{l,\alpha },\ \ \lim _{n\rightarrow \infty }x_{n,r\alpha }=x_{r,\alpha }, \end{aligned}$$

Namely,

$$\begin{aligned} \lim _{n\rightarrow \infty }D(x_n,x)=\lim _{n\rightarrow \infty }\sup _{\alpha \in (0,1]}\{\max \{|x_{n,l,\alpha }-x_{l,\alpha }|, |x_{n,r,\alpha }-x_{r,\alpha }|\}\}=0. \end{aligned}$$

This completes the proof of Theorem 3.3. $\square$

Theorem 3.3 describes the development process error item may much less than the population threshold density as (3.29), when the initial fuzzy size meet Case I of the FBQP (2).

**Secondly, if Case II occurs,** it follows that, for $\alpha \in (0,1], n=0,1,2,\cdots ,$

34 $$\begin{aligned}{} & {} x_{n+1,l,\alpha }=A_{l,\alpha }+B_{l,\alpha }x_{n,l,\alpha }+\frac{x_{n,r,\alpha }}{x_{n-1,r,\alpha }x_{n-2,r,\alpha }},\nonumber \\{} & {} x_{n+1,r,\alpha }=A_{r,\alpha }+B_{r,\alpha }x_{n,r,\alpha }+\frac{x_{n,l,\alpha }}{x_{n-1,l,\alpha }x_{n-2,l;,\alpha }}. \end{aligned}$$

To obtain the dynamical behavior of Eq. (2) in Case II as (3.3) , we need the following lemma.

#### Lemma 3.5

Consider the system of difference equations

35 $$\begin{aligned} y_{n+1}=p+ay_n+\frac{z_n}{z_{n-1}z_{n-2}},\ \ z_{n+1}=q+bz_n+\frac{y_n}{y_{n-1}y_{n-2}},\ \ n=0,1,\cdots , \end{aligned}$$

if $0<a<1,~0<b<1, y_{i}>0, z_{i}>0, i=-2,-1,0$. Then, for $n\ge 1,$

36 $$\begin{aligned} p\le y_n\le \frac{c_1}{1-a}+y_2,\ \ q\le z_n\le \frac{c_2}{1-b}+z_2, \end{aligned}$$

where

$$\begin{aligned} c_1=p+\frac{1+b}{q}+\frac{1+a}{q^2p}+ \frac{1}{q^3p^2},\ \ c_2=q+\frac{1+a}{p}+\frac{1+b}{p^2q}+ \frac{1}{p^3q^2}. \end{aligned}$$

#### Proof

From (3.32), for $n\ge 1,$ it is clear that $y_n\ge p, z_n\ge q.$ And for $n\ge 4$,

$$\begin{aligned} y_{n+1}= & {} p+ ay_{n} + \frac{z_{n}}{z_{n-1}z_{n-2}}= p+ ay_{n} + \frac{q+bz_{n-1}+\frac{y_{n-1}}{y_{n-2}y_{n-3}}}{z_{n-1}z_{n-2}} \nonumber \\= & {} p+ ay_{n} + \frac{q}{z_{n-1}z_{n-2}}+ \frac{b}{z_{n-2}}+ \frac{p}{z_{n-1}z_{n-2}{y_{n-2}y_{n-3}}}+ \frac{a}{z_{n-1}z_{n-2}y_{n-3}}+ \frac{1}{z_{n-1}z_{n-3}z_{n-4}y_{n-2}y_{n-3}} \nonumber \\\le & {} ay_{n}+p+ \frac{q}{q^2}+ \frac{b}{q}+ \frac{p}{q^2p^2}+ \frac{a}{q^2p}+ \frac{1}{q^3p^2} \nonumber \\= & {} ay_{n}+c_1. \end{aligned}$$

$$\square $$

Similarly,

$$\begin{aligned} z_{n+1}\le bz_n+c_2. \end{aligned}$$

By recursive method, one can get that

$$\begin{aligned} \left\{ \begin{array}{lll} y_{n+1} &{}\le a(ay_n+c_1)+c_1\le \dots \le a^{n}y_2+\frac{c_1(1-a^{n-1})}{1-a}\le y_2+\frac{c_1}{1-a}, \\ &{}&{}\\ z_{n+1} &{}\le b(bz_n+c_2)+c_2\le \dots \le b^{n}z_2+\frac{c_2(1-b^{n-1})}{1-b}\le z_2+\frac{c_2}{1-b}, \end{array} \right. \end{aligned}$$

This completes the proof of Lemma 3.5.

#### Theorem 3.4

Consider Eq. (2), where the parameters $A, B\in R_F^+$ and the initial conditions $x_i\in R_F^+, i=-2, -1, 0$. If

37 $$\begin{aligned} \left\{ \begin{array}{l} B_{r,\alpha }\le B_{r,0}< 1,\\ \\ x_{n,l,\alpha }x_{n-1,r,\alpha }x_{n-2,r,\alpha } > x_{n,r,\alpha }x_{n-1,l,\alpha }x_{n-2,l,\alpha }, \end{array} \right. \end{aligned}$$

for $\alpha \in (0,1]$, then every positive fuzzy solution $x_n$ of Eq. (2) is bounded and persistent.

#### Proof

Set $C=(C_{l,\alpha },C_{r,\alpha }), \alpha \in (0,1],$ the proof process is similar to Theorem 3.2. With (3.33) in Lemma3.5,

$$\begin{aligned}{}[x_{n,l,\alpha },x_{n,r,\alpha }] \subset [A_{l,\alpha }~,~~x_{2,l,\alpha }+\frac{C_{l,\alpha }}{1-B_{l,\alpha }}] \times [A_{r,\alpha }~,~~x_{2,r,\alpha }+\frac{C_{r,\alpha }}{1-B_{r,\alpha }}], \end{aligned}$$

where

$$\begin{aligned} C_{l,\alpha }=A_{l,\alpha }+ \frac{1+B_{r,\alpha }}{A_{r,\alpha }}+ \frac{1+B_{l,\alpha }}{A_{r,\alpha }^2A_{l,\alpha }}+\frac{1}{A_{r,\alpha }^3A_{l,\alpha }^2},~~ C_{r,\alpha }=A_{r,\alpha }+ \frac{1+B_{l,\alpha }}{A_{l,\alpha }}+ \frac{1+B_{r,\alpha }}{A_{l,\alpha }^2A_{r,\alpha }}+\frac{1}{A_{l,\alpha }^3A_{r,\alpha }^2}. \end{aligned}$$

$$\square $$

We completes the proof that the positive fuzzy solution $x_n$ is bounded and persistent.

Theorem 3.4 reveals the sufficient condition for a fuzzy Bobwhite quail population steady, in Case II, is only related to the population initial size and its threshold density.

#### Lemma 3.6

Consider the system of difference equations (3.32), if

38 $$\begin{aligned} \left\{ \begin{array}{l} 0<a<1,0<b<1,\\ \\ \frac{p^2}{1-a}<\frac{q^2}{1-b}, \end{array} \right. \end{aligned}$$

then there exists the unique positive equilibrium $({\widetilde{y}},{\widetilde{z}}),$ which

39 $$\begin{aligned} ({\widetilde{y}},{\widetilde{z}})=\left( \frac{pq-(b-a)+\sqrt{(pq+a-b)^2+4pq(1-a)}}{2q(1-a)}, \frac{pq+(b-a)+\sqrt{(pq+b-a)^2+4pq(1-b)}}{2p(1-b)}\right) \end{aligned}$$

is asymptotically stable.

#### Proof

According (3.32), its equilibrium should meet

$$\begin{aligned} {\widetilde{y}}=p+a {\widetilde{y}}+\frac{1}{{\widetilde{z}}},\ \ {\widetilde{z}}=q+b{\widetilde{z}}+\frac{1}{ {\widetilde{y}}},\ \ n=0,1,\cdots , \end{aligned}$$

It is easy to obtain the unique positive equilibrium point $({\widetilde{y}},{\widetilde{z}})$ with expression in (3.36). $\square$

We have the series partial derivatives of $y_n, z_n$ to the recording delayed values $y_{n-1},y_{n-2},z_{n-2}$ and $z_{n-2}$ for $n=0,1,\cdots$ as

$$\begin{aligned}{} & {} \frac{\partial y_{n+1}}{\partial y_n}=a,\ \ \frac{\partial y_{n+1}}{\partial z_n}=\frac{1}{z_{n-1}z_{n-2}},\ \ \frac{\partial y_{n+1}}{\partial z_{n-1}}=-\frac{z_n}{z_{n-1}^2z_{n-2}},\ \ \frac{\partial y_{n+1}}{\partial z_{n-2}}=-\frac{z_n}{z_{n-1}z_{n-2}^2},\\{} & {} \frac{\partial z_{n+1}}{\partial z_n}=b,\ \ \frac{\partial z_{n+1}}{\partial y_n}=\frac{1}{y_{n-1}y_{n-2}},\ \ \frac{\partial z_{n+1}}{\partial y_{n-1}}=-\frac{y_n}{y_{n-1}^2y_{n-2}},\ \ \frac{\partial z_{n+1}}{\partial y_n}=-\frac{y_n}{y_{n-1}y_{n-2}^2},\ \ \end{aligned}$$

The linearized equation of system (3.32) about $({\widetilde{y}},{\widetilde{z}})$ is

$$\begin{aligned} \Psi _{n+1}=T\Psi _n \end{aligned}$$

where $$\Psi _n=(y_n,y_{n-1},y_{n-2},z_n,z_{n-1},z_{n-2})^T$$, and

$$\begin{aligned} T=\left( \begin{array}{cccccc} a&{} 0&{} 0&{} 1/{\widetilde{z}}^2 &{}-1/{\widetilde{z}}^2&{}-1/{\widetilde{z}}^2\\ 1&{} 0 &{}0 &{}0 &{}0 &{}0 \\ 0&{} 1 &{}0 &{}0 &{}0 &{}0\\ 1/{\widetilde{y}}^2&{} -1/{\widetilde{y}}^2&{}-1/{\widetilde{y}}^2&{}b&{} 0&{} 0\\ 0&{}0&{}0&{}1&{}0&{}0\\ 0&{}0&{}0&{}0&{}1&{}0 \end{array} \right) \end{aligned}$$

Let $G=\text{ diag }(1,\varepsilon ^{-1},\varepsilon ^{-2},\cdots ,\varepsilon ^{-5})$ be a diagonal matrix, let

$$\begin{aligned} \root 6 \of {\left( \frac{1-b}{1-a}\right) \frac{p^{2}}{q^{2}}}<\varepsilon <1. \end{aligned}$$

Clearly, *G* is invertible. Computing $GTG^{-1}$, we have

$$\begin{aligned} L=GTG^{-1}= \left( \begin{array}{cccccc} a&{}0&{}0&{}1/\varepsilon ^{3}{\widetilde{z}}^2&{}-1/\varepsilon ^{4}{\widetilde{z}}^2&{}-1/\varepsilon ^{5}{\widetilde{z}}^2\\ \varepsilon &{}0&{}0&{}0&{}0&{}0\\ 0&{}\varepsilon &{}0&{}0&{}0&{}0\\ \varepsilon ^{3}/{\widetilde{y}}^2&{}-\varepsilon ^{2}/{\widetilde{y}}^2&{}-\varepsilon /{\widetilde{y}}^2 &{}b&{}0&{}0\\ 0&{}0&{}0&{}\varepsilon &{}0&{}0\\ 0&{}0&{}0&{}0&{}\varepsilon &{}0 \end{array} \right) . \end{aligned}$$

From (3.35), we know

$$\begin{aligned} \Vert L\Vert _{\infty }=\Vert GTG^{-1}\Vert _{\infty }=\max _{1\le i\le 6}\left\{ \sum _{j=1}^{6}|L_{ij}|\right\} <1. \end{aligned}$$

By , it follows the positive equilibrium $({\widetilde{y}},{\widetilde{z}})$ is asymptotically stable.

#### Lemma 3.7

Consider the system of difference equations (3.32), if

40 $$\begin{aligned} \left\{ \begin{array}{l} 0<a<1,0<b<1,\\ \\ (1-b)p^2<(1-a)q^2,\\ \\ 1 < (1-a)(1-b)p^{2}q^{2}, \end{array} \right. \end{aligned}$$

hold true, then every positive solution $(y_n,z_n)$ of (3.32) tends to the equilibrium $({\widetilde{y}},{\widetilde{z}}).$

#### Proof

Suppose that $(y_n,z_n)$ is an arbitrary positive solution of (3.32). Set

$$\begin{aligned} \lim _{n\rightarrow \infty }\sup y_n=L_1,\lim _{n\rightarrow \infty }\inf y_n=l_1,\ \ \lim _{n\rightarrow \infty }\sup z_n=L_2,\ \ \ \lim _{n\rightarrow \infty }\inf z_n=l_2. \end{aligned}$$

where $$l_i, L_i\in (0,+\infty ), i=1,2.$$ Then

$$\begin{aligned} L_1\le p+aL_1+\frac{L_2}{{\widetilde{z}}l_2}, \ \ l_1\ge p+al_1+\frac{l_2}{{\widetilde{z}}L_2},\ \ \ L_2\le q+bL_2+\frac{L_1}{{\widetilde{y}}l_1}, \ \ l_2\ge q+bl_2+\frac{l_1}{{\widetilde{y}}L_1}, \end{aligned}$$

where $({\widetilde{y}},{\widetilde{z}})$ is the positive equilibrium of (3.32). Then

41 $$\begin{aligned} \left[ (1-a)(1-b)-1/~{\widetilde{y}}^2{\widetilde{z}}^2\right] (L_2-l_2)(L_1-l_1)\le 0. \end{aligned}$$

From (3.32), (3.37) and (3.38) it can follow that $L_i=l_i, i=1,2.$ Therefore,

$$\begin{aligned} \lim _{n\rightarrow \infty }y_{n}={\widetilde{y}},\ \ \lim _{n\rightarrow \infty }z_{n}={\widetilde{z}}. \end{aligned}$$

The proof of Lemma 3.7 is completed. $\square$

Combining Lemma 3.6 with Lemma 3.7, we know Eq. (2) globally asymptotically stable with fuzzy equilibrium solution *x* as the following theorem.

#### Theorem 3.5

Consider Eq. (2), if the following conditions hold true for $\alpha \in (0,1],$

42 $$\begin{aligned} \left\{ \begin{array}{l} B_{r,\alpha }\le B_{r,0}<1,\\ \\ (1-B_{r,\alpha })A_{l,\alpha }^{2}<(1-B_{l,\alpha })A_{r,\alpha }^{2},\\ \\ 1<(1-B_{l,\alpha })(1-B_{r,\alpha })A_{l,\alpha }^{2}A_{r,\alpha }^{2}, \\ \\ x_{n,l,\alpha }x_{n-1,r,\alpha }x_{n-2,r,\alpha } > x_{n,r,\alpha }x_{n-1,l,\alpha }x_{n-2,l,\alpha }, \end{array} \right. \end{aligned}$$

Then there exists a unique positive fuzzy equilibrium *x*, where $[x]_\alpha =[x_{l,\alpha }, x_{r,\alpha }],$

43 $$\begin{aligned} \begin{array}{l} x_{l,\alpha }=\frac{B_{l,\alpha }-B_{r,\alpha }+A_{l,\alpha }A_{r,\alpha } +\sqrt{(B_{l,\alpha }-B_{r,\alpha }+A_{l,\alpha }A_{r,\alpha })^2+4A_{l,\alpha } A_{r,\alpha }(1-B_{l,\alpha })}}{2A_{r,\alpha }(1-B_{l,\alpha })}, \\ \\ x_{r,\alpha }=\frac{B_{r,\alpha }-B_{l,\alpha }+A_{l,\alpha }A_{r,\alpha } +\sqrt{(B_{r,\alpha }-B_{l,\alpha }+A_{l,\alpha }A_{r,\alpha })^2+4A_{l,\alpha } A_{r,\alpha }(1-B_{r,\alpha })}}{2A_{l,\alpha }(1-B_{r,\alpha })}. \end{array} \end{aligned}$$

and $$\lim _{n\rightarrow \infty }D(x_n,x)=0.$$

#### Proof

Assume there is a fuzzy number *x* satisfying

$$\begin{aligned}{} & {} x_{l,\alpha }=A_{l,\alpha }+B_{l,\alpha }x_{l,\alpha }+\frac{x_{r,\alpha }}{x_{r,\alpha }^2},\\{} & {} x_{r,\alpha }=A_{r,\alpha }+B_{r,\alpha }R_\alpha +\frac{x_{l,\alpha }}{x_{l,\alpha }^2}. \end{aligned}$$

From which, we have (3.40).

Let $x_n$ be a positive solution of (2). Since (3.39) is satisfied, by virtue of Lemma 3.6 and Lemma 3.7, we have

$$\begin{aligned}{} & {} \lim _{n\rightarrow \infty }x_{n,l,\alpha }=x_{l,\alpha },\\{} & {} \lim _{n\rightarrow \infty }x_{n,r,\alpha }=x_{r,\alpha }. \end{aligned}$$

Then

$$\begin{aligned}{} & {} \lim _{n\rightarrow \infty }D(x_n,x)=\lim _{n\rightarrow \infty }\sup _{\alpha \in (0,1]}\{\max \{|x_{n,l,\alpha }-x_{l,\alpha }|, |x_{n,r,\alpha }-x_{r,\alpha }|\}\}=0. \end{aligned}$$

The proof of Theorem 3.5 is completed. $\square$

Theorem 3.5 revelates the relationship between the error threshold item and the population threshold density of FBQP model (2), when the initial fuzzy size meets Case II. Condition (3.39) is necessary to condition (3.29).

## Numerical examples

### Example 4.1

For reversing the quail decline in Texas, the State Wildlife Department and Texas A & M Agrilife Extension Service had funded a series of research and investigations, such as mentioned in^24^, which scaled quail and bobwhite in the North Texas by a pairwise sequentially Markovian coalescent (PSMC) model (^47^).

Considering the inevitable error during data acquisition and prepossessing, with incompleteness of living environment parameters, we belive the FBQP model may depict and evolve the population of quail bobwhite more practically, rather than PSMC model. We set the parameters *A* with median 1.75 in (2), for the stable governmental input on agricultural resources, and *B* with median 0.15 for the reason that quail bobwhite had a pessimistic natural growth rate in situation in those years.

Furthermore, the preceding population structure with three periods is an appropriate delay for the biotic population. For simplicity, first of the population size are set to be a unit with cumulative fuzzy degrees as following,

44 $$\begin{aligned} \left\{ \begin{array}{l} \overline{\bigcup _{\alpha \in (0,1]}[A]_\alpha }=[1.5,2],\ \ \overline{\bigcup _{\alpha \in (0,1]}[B]_\alpha }=[0.15,0.35],\\ \\ \overline{\bigcup _{\alpha \in (0,1]}[x_{-2}]_\alpha }=[0.75,1.25], \ \ \overline{\bigcup _{\alpha \in (0,1]}[x_{-1}]_\alpha }=[0.65,1.35],\ \ \overline{\bigcup _{\alpha \in (0,1]}[x_{0}]_\alpha }=[0.55,1.45]. \end{array} \right. \end{aligned}$$

From (4.1), the corresponding fuzzy parameters and initial values are expressed in Parabolic fuzzy numbers(PFNs) as mentioned in^35–38^ to depict fuzzy phenomenons, special expressing the system and period efficiencies of non-performing assets in^48^, where the degree of fuzzy $\alpha \in (0,1],$

45 $$\begin{aligned} \left\{ \begin{array}{lll} \left[ A\right] _{\alpha }&{}=&{}\left[ {1.75-0.25*\sqrt{1-\alpha }, 1.75+0.25*\sqrt{1-\alpha }}\right] ,\\ &{}&{}\\ \left[ B\right] _{\alpha }&{}=&{}\left[ {0.25-0.1*\sqrt{1-\alpha }, 0.25+0.1*\sqrt{1-\alpha }}\right] ,\\ &{}&{}\\ \left[ x_{-2}\right] _\alpha &{}=&{}\left[ 1-0.25*\sqrt{1-\alpha },1+0.25*\sqrt{1-\alpha }\right] ,\\ &{}&{}\\ \left[ x_{-1}\right] _\alpha &{}=&{}\left[ 1-0.35*\sqrt{1-\alpha },1+0.35*\sqrt{1-\alpha }\right] ,\\ &{}&{}\\ \left[ x_{0}\right] _\alpha &{}=&{}\left[ 1-0.45*\sqrt{1-\alpha },1+0.45*\sqrt{1-\alpha }\right] . \end{array} \right. \end{aligned}$$

The parabolic fuzzy numbers are functions according to $\alpha$, which brings out simulation with Matlab with expression as following,

46 $$\begin{aligned} \left\{ \begin{array}{ll} A(x)={1-16(x-1.75)^2, \quad 1.5 \le x \le 2}; \\ \\ B(x)={1-100(x-0.25)^2, \quad 0.15 \le x \le 0.35}; \\ \\ x_{-2}(x)=1-16(x-1)^2, \quad \ 0.5 \le x \le 1.5;\\ \\ x_{-1}(x)=1-\frac{200}{49}(x-1)^2, \quad \ 0.65\le x \le 1.35;\\ \\ x_0(x)=1-\frac{200}{81}(x-1)^2, \quad \ 0.55\le x \le 1.45; \end{array} \right. \end{aligned}$$

**Figure 1:**
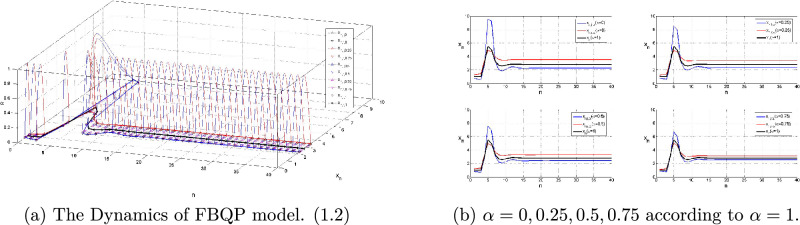
An Example of FBQP model. (2) in Case I.

The FBQP model (2):

$$\begin{aligned} x_{n+1}=A+Bx_n+\frac{x_n}{x_{n-1}x_{n-2}},\ \ n=0,1,\cdots , \end{aligned}$$

with (4.2) fits both Case I and Case II in the method of fuzzy g-division. Based on Theorem 3.2 and Theorem 3.3, model (2) have stable evolution ultimately in Table 1 and numerical simulation diagram in Fig. 1.

**Table 1:** FBQP model.(2) in Case I, where $A,B,x_{-2},x_{-1},x_{0}$ are fuzzy parameters in (4.2)

	$A_{r}$	$B_{r}$	$x_{-2,r}$	$x_{-1,r}$	$x_{0,r}$	$x_r$	$x^*_{r}$
	$A_{l}$	$B_{l}$	$x_{-2,l}$	$x_{-1,l}$	$x_{0,l}$	$x_l$	$x^*_{l}$
$\alpha$=0	2.0000	0.3500	1.2500	1.3500	1.4500	(2.0000,6.4500)	3.5147
	1.5000	0.1500	0.7500	0.6500	0.5500	(1.5000,4.4486)	2.2806
$\alpha$=0.25	1.9665	0.3366	1.2165	1.3031	1.3897	(1.9665,6.2476)	3.4068
	1.5335	0.1634	0.7835	0.6969	0.6103	(1.5335,4.5114)	2.3431
$\alpha$=0.5	1.9268	0.3207	1.1768	1.2475	1.3182	(1.9268,6.0185)	3.2846
	1.5732	0.1793	0.8232	0.7525	0.6818	(1.5732,4.5989)	2.4203
$\alpha$=0.75	1.8750	0.3000	1.1250	1.1750	1.2250	(1.8750,5.7370)	3.1344
	1.6250	0.2000	0.8750	.82500	0.7750	(1.6520,4.7321)	2.5261
$\alpha$=1	1.7500	0.2500	1.0000	1.0000	1.0000	(1.7500,5.1325)	2.8081
	1.7500	0.2500	1.0000	1.0000	1.0000	(1.7500,5.1325)	2.8081

The fuzzy positive solution is proved to be bounded and persistent that $x_n \in (x_{l,\alpha },x_{r,\alpha })=(A_{l,\alpha },\frac{A_{r,\alpha }^3}{(1-B_{r,\alpha })A_{l,\alpha }^2-1}+x_{3,r,\alpha })$, and its fuzzy equilibrium solution is $(x^*_{l,\alpha },x^*_{r,\alpha })$ as the following table shows.

Obviously, every fuzzy $x_n$ of FBQP model.(2) tends to the unique fuzzy equilibrium $x^*$ with respect to *D* as $n\rightarrow \infty$, see Fig. 1.

**Figure 2:**
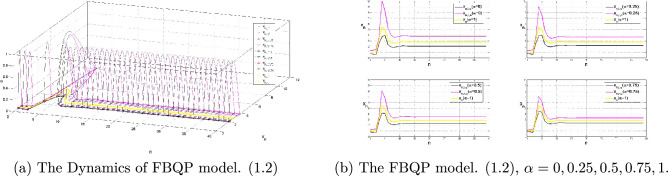
An Example of fuzzy Bobwhite quail populations Model in Case II.

Based on Theorem 3.4 and Theorem 3.5, model (2) have stable evolution ultimately in Table 2 and numerical simulation diagram in Fig. 2.

**Table 2:** FBQP model.(2) with (4.2)

	$A_{r}$	$B_{r}$	$x_{-2,r}$	$x_{-1,r}$	$x_{0,r}$	$x_r$	$x^*_{r}$
	$A_{l}$	$B_{l}$	$x_{-2,l}$	$x_{-1,l}$	$x_{0,l}$	$x_l$	$x^*_{l}$
$\alpha$=0	2.0000	0.3500	1.2500	1.3500	1.4500	(2.0000,6.2819)	3.8192
	1.5000	0.3366	0.7500	0.6500	0.5500	(1.5000,3.3997)	2.0728
$\alpha$=0.25	1.9665	0.2433	1.2165	1.3031	1.3897	(1.9665,6.0414)	4.6126
	1.5335	0.1567	0.7835	0.6969	0.6103	(1.5335,3.5571)	2.1594
$\alpha$=0.5	1.9268	0.3207	1.1768	1.2475	1.3182	(1.9268,5.7677)	3.4860
	1.5732	0.1793	0.8232	0.7525	0.6818	(1.5732,3.7487)	2.2664
$\alpha$=0.75	1.8750	0.3000	1.1250	1.1750	1.2250	(1.8750,5.4283)	3.2705
	1.6250	0.2000	0.8750	0.8250	0.7750	(1.6250,4.0073)	2.4135
$\alpha$=1	1.7500	0.2500	1.0000	1.0000	1.0000	(1.7500,4.6779)	2.8081
	2.0000	1.7500	1.0000	1.0000	1.0000	(1.7500,4.6779)	2.8081

The fuzzy positive solution have a similar presentation as in Case I, it is bounded and persistent that $x_n \in (x_{l,\alpha },x_{,\alpha })=(A_{l,\alpha },A_{r,\alpha }+ \frac{1+B_{l,\alpha }}{A_{l,\alpha }}+ \frac{1+B_{r,\alpha }}{A_{l,\alpha }^2A_{r,\alpha }}+\frac{1}{A_{l,\alpha }^3A_{r,\alpha }^2})$ as the following table shows.

Furthermore every positive solution $x_n$ of Eq. (2) tends to the unique fuzzy equilibrium $x^*$ with respect to *D* as $n\rightarrow \infty$, see Fig. 2.

**Figure 3:**
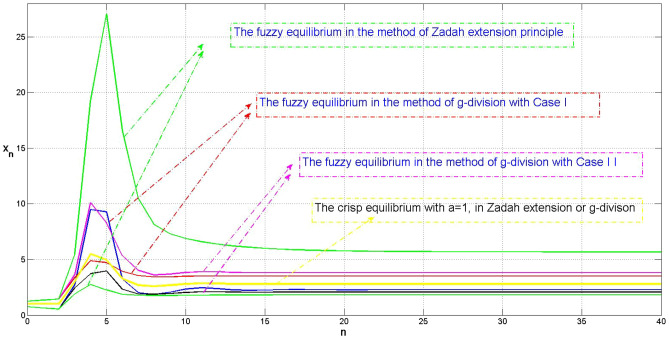
The comparison of fuzzy equilibrium in g-division and Zadah extension principle with the crisp equilibrium.

In Fig. 3, we compared the evolution of model (2) with (4.2) in method of classic division (Zadah extension principle) and general division (g-division) in Case I and Case II, with the maximum degree of ambiguity ($\alpha =0$). Meanwhile, the crisp model evolution ($\alpha =1$) is arranged to demonstrate the relationship between fuzzy solutions with crisp solution.

### Example 4.2

**Figure 4:**
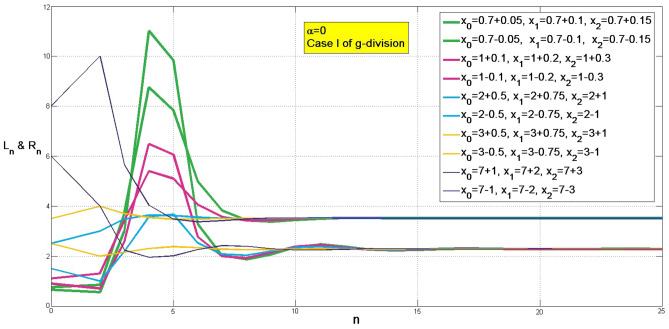
A demonstration of eventual stability of FBQP model (2) with *A*, *B* in (4.2), in Case I of g-division.

Accounting FBQP model.(2) constitutionally, we say it is an anti-example to Allee effect not only from the analysis in Theorem3.3 and Theorem 3.5, but also from the phenomenon with several initial population, higher or lower than the unit. Without losing the generation, Fig. 4 demonstrates a FBQP model with *A*, *B* in (4.2).

As a matter of fact, it has a similar line-trend in Case II. We hope these phenomenon with wide original value range may convince one that Allee effect will not work in the thematic model.

## Conclusion and postscript

We applied an uncertainty analysis with fuzzy degree to anticipate some species and organism in surrounding with vagueness and uncertainty,studied a class of fuzzy Bobwhite quail populations model.

In fact, the results interpreted some ecological population experiment. For example, a research group from Colorado-state University trapped and translocated quail from source populations to a large contiguous release site in Knox County, Texas, USA during 2016-2017, as in^49^. They evaluated mortality and dispersal of these individuals by using a multi-state mark-recapture model with state uncertainty. Our results served Ruzicka’s conclusions that the population size difference in mortality and dispersal was the largest effect potentially and is likely attributable to weather conditions in seasons. More compatible findings are reflected in several recent relevant research,as in^50^, and^51^so on.

### Supplementary Information

Supplementary Information.

## Data Availability

The datasets used and/or analyzed during the current study are available from the first author on reasonable request.
