# Supplementary material for: Dynamic analysis of a fuzzy Bobwhite quail population model under g-division law
Source: Sci Rep. 2024 Apr 27;14:9682. doi: 10.1038/s41598-024-60178-4 (PMC11055902; doi:10.1038/s41598-024-60178-4)
Supplement: Supplementary file 1 — Supplementary Information. [file 41598_2024_60178_MOESM1_ESM.docx]

Data1.m

clear all % Case I

a=0 % \alpha =0,0.25,0.5,0.75,1

AL=1.75-0.25.*sqrt(1-a) ;

AR=1.75+0.25.*sqrt(1-a);

BL=0.25-0.1.*sqrt(1-a) ;

BR=0.25+0.1.*sqrt(1-a);

x(1)=1-(0.25).*sqrt(1-a);

y(1)=1+(0.25).*sqrt(1-a);

x(2)=1-(0.35).*sqrt(1-a);

y(2)=1+(0.35).*sqrt(1-a);

x(3)=1-(0.45).*sqrt(1-a);

y(3)=1+(0.45).*sqrt(1-a);

for n=1:41

x(n+3)=AL+BL.*x(n+2)+(x(n+2))./(x(n+1).*x(n));

y(n+3)=AR+BR.*y(n+2)+(y(n+2))./(y(n+1).*y(n));

end

xmin=AL;

X=[x(1),x(2),x(3)] ;

Y=[y(1),y(2),y(3)];

xmax=AL^3/((1-BL)*AL^2-1)+max(X);

ymin=AR;

ymax=AR^3/((1-BR)*AR^2-1)+max(Y);

x(44)

y(44)

Data2

clear % Case II

a=0 % \alpha =0,0.25,0.5,0.75,1

AL=1.75-0.25.*sqrt(1-a) ;

AR=1.75+0.25.*sqrt(1-a);

BL=0.25-0.1.*sqrt(1-a) ;

BR=0.25+0.1.*sqrt(1-a);

x(1)=1-(0.25).*sqrt(1-a);

y(1)=1+(0.25).*sqrt(1-a);

x(2)=1-(0.35).*sqrt(1-a);

y(2)=1+(0.35).*sqrt(1-a);

x(3)=1-(0.45).*sqrt(1-a);

y(3)=1+(0.45).*sqrt(1-a);

for n=1:41

x(n+3)=AL+BL.*x(n+2)+(y(n+2))./(y(n+1).*y(n));

y(n+3)=AR+BR.*y(n+2)+(x(n+2))./(x(n+1).*x(n));

end

X=[x(1),x(2),x(3)]

Y=[y(1),y(2),y(3)]

xmin=AL

xmax=(AL+(1+BR)/(AR)+(1+BL)/(AR^2*AL)+ 1/(AR^3*AL^2))/(1-BL) +x(3)

ymin=AR

ymax=(AR+(1+BL)/(AL)+(1+BR)/(AL^2*AR)+ 1/(AL^3*AR^2))/(1-BR)+y(3)

x(44)

y(44)
